# Assessment of Background Illumination Influence on Accuracy of Measurements Performed on Optical Coordinate Measuring Machine Equipped with Video Probe

**DOI:** 10.3390/s21072509

**Published:** 2021-04-03

**Authors:** Wiktor Harmatys, Adam Gąska, Piotr Gąska, Maciej Gruza, Jerzy A. Sładek

**Affiliations:** Laboratory of Coordinate Metrology, Cracow University of Technology, al. Jana Pawła II 37, 31-864 Kraków, Poland; wiktor.harmatys@pk.edu.pl (W.H.); piotr.gaska@pk.edu.pl (P.G.); maciej.gruza@pk.edu.pl (M.G.); jerzy.sladek@pk.edu.pl (J.A.S.)

**Keywords:** video probe, optical coordinate measuring machine, illumination, accuracy

## Abstract

Currently the Coordinate Measuring Technique is facing new challenges both in terms of used methodology and a speed of measurement. More and more often modern optical systems or multisensor systems replace classic solutions. Measurement performed using the optical system is more vulnerable to incorrect points acquisition due to such factors as an inadequate focus or parameters of applied illumination. This article examines the effect of an increasing illumination on the measurement result. A glass reference plate with marked circles and a hole plate standard were used for the measurements performed on a multi-sensor machine Zeiss O’ Inspect 442. The experiment consisted of measurements of standard objects with different values of the backlight at the maximum magnification. Such approach allows to assess the influence of controlled parameter on errors of diameter and form measurements as well as an uncertainty of measurements by determination of ellipses of point repeatability. The analysis of the obtained results shows that increasing backlight mainly affects the result of the diameter measurement.

## 1. Introduction

Coordinate Metrology is a field of metrology with a very wide range of applications. All objects in the space that surrounds us are three-dimensional. Most of them are manufactured according to the shape previously designed in accordance with the technical documentation. Such items in the documentation are described as a view or a section along with any requirements for their execution, basic dimensions, tolerances, etc. Information about the form and individual dimensions of the measured object in coordinate measuring technique is perceived as a set of coordinates of points. For the measurements, coordinate machines are used, which currently can be divided into two groups: contact machines and optical machines. The oldest and most numerous group is constituted by devices that use contact probe heads in order to obtain the coordinates of the contact point with a measured surface. Such machines have been developed since the second half of 20th century, and their properties have been thoroughly studied and described in literature. However, over the last years, the second of mentioned groups is gaining more and more attention. Contactless coordinate machines offer some advantages which are virtually inaccessible to tactile systems, mainly incomparable speed of points coordinates acquisition. At the same time, accuracy of contactless systems is described generally as lower than for contact coordinate measuring machines (CMMs). The solution that efficiently combines the advantages of classical coordinate machines and optical machines is a group of so-called multisensor machines [1,2]. As a relatively new solution, such systems require intensive research, especially on the accuracy of measurements [3,4].

The main contributors to overall accuracy of machines of this kind include sources connected with machine kinematics and tactile probe characteristics, which can be tested and described in similar manner as in case of classic CMMs. Additionally, sources related with utilization of different contactless probing systems can be pointed out such as: errors connected with a camera or a white light sensor functioning, algorithms used for edges detection or properties of applied lighting.

Topic of an illumination influence on the accuracy of measurements performed using optical coordinate measuring machines (OCMM) equipped with a video probe was rarely investigated in the past. In [5] Kim and McKeown presented analytical and experimental approaches to exploring how the measuring uncertainty limit of video probes is determined by major design parameters, one of them being the illumination. Using an example of optimal design, they demonstrated that an ultraprecision measurement of 0.01 μm uncertainty can be practically achieved providing optimal lighting conditions. In [6], Tran and Claudet reported on the effect of the sensitivity of vision probing on an OCMM to different lighting conditions, both for unidirectional and bidirectional measurements. They found that the lighting is a major contributor to the measurement error budget, especially when a bidirectional measurement needs to be made.

There are also some papers treating generally about an issue of measurement accuracy and the uncertainty in measurements performed using optical CMMs in which the illumination is considered as one of uncertainty contributors. In [7], Carmignato and others used two artefacts that are commonly used for performance verification of optical CMMs: a linear glass scale and an optomechanical hole plate for quantification of uncertainty contributors in coordinate measurements using video probes. The results showed the significant influence of illumination, an objective magnification, a measuring window size, use of autofocus and an image filtering on measurement uncertainty. In [3], Weckenmann and Bernstein described a prototype of an optical multi-sensor-measurement system combining a shadow system and a light-section system for the in-line inspection of concave extruding profiles. Experimental analysis of a measurement uncertainty was performed. To this aim, the effect of typical environmental influences like a dust, object’s vibrations, the illuminations’ pitch error or extraneous light on the measurement accuracy was examined. After those analyses, the measurement system was evaluated under shop floor conditions. Authors found that the influences of an extraneous light and reflections are the reasons for the higher uncertainty values. In [8], Carmignato presented an industrial comparison of CMMs equipped with optical sensors, performed in Europe through 1.5-year time period. Participants were chosen mainly from small-medium size industrial companies. On each CMM taking part in this comparison, a set of calibrated artefacts (glass scale, optomechanical hole plate and 3D injection molded standards) with measurement tasks of different complexity was measured. In addition to the evaluation of actual metrological performances of optical CMMs in industry; also, an uncertainty estimation was performed. One of the error sources demonstrated by the results was the use of translucent materials (such as plastic) that can transmit or reflect the light depending on the material properties (e.g., color) and on the type of light used, causing an effect of shrinkage or distortion of the measured features. Other papers treating accuracy and uncertainty in optical measurements may be found in [9,10,11,12].

Fundamental information on optics used in dimensional metrology and error sources including lighting conditions are given in [13,14,15,16]. Schwenke et al. provided a technical overview of the optical methods available for the dimensional metrology in [17]. Methods for the measurement of length, an angle, a surface form and spatial coordinates were described. In the paper, both the metrological characteristics and the technical limitations of the methods were presented along with some new and promising approaches that may play an important role in the dimensional metrology for production. Moreover, an influence of illumination-related factors like an ambient light, refraction effects, an out-of-focus blur and other is investigated. In [18], Larue analyzed all the factors that go into measurement precision with the illumination as one of them and showed how the use of optical technologies makes it possible to greatly reduce the primary causes of measurement imprecision. The illustration of such usages was also given in the form of specific cases taken from real applications in the aeronautical, automotive, and naval industries.

Another group of papers that investigate lighting influence on optical coordinate measurements are publications treating about sensors performance in multisensor systems and data fusion techniques used for data obtained with their use. Examples of such research may be found in [19,20,21,22,23].

Works on development of a virtual model of optical CMM equipped with the video probe are now in the final stage in Laboratory of Coordinate Metrology. This model will be based on a description of the measurement uncertainty using multiple simulations of measuring points reproduction expressed as repeatability ellipses [24]. In order to make the virtual model fully functional, it is necessary to know two things:

1. Values of task-specific error changes related to measurement of distances, positions and different form deviations under changing lighting conditions (needed for systematic error correction of single measurement result).
2. Changes in uncertainty areas of a measuring point reproduction for point measurements under changing lighting conditions (needed for simulation of uncertainty associated to single measurement).

Investigations presented in this paper aim to determine the abovementioned changes introduced by changes in the backlight illumination of the measured workpiece.

The rest of the paper is arranged in the following way: Section 2 describes the methodology used in experiments; obtained results are shown in Section 3, while Section 4 includes a discussion over the experiments results and presents the direction of future works.

## 2. Materials and Methods

**Figure 1:**
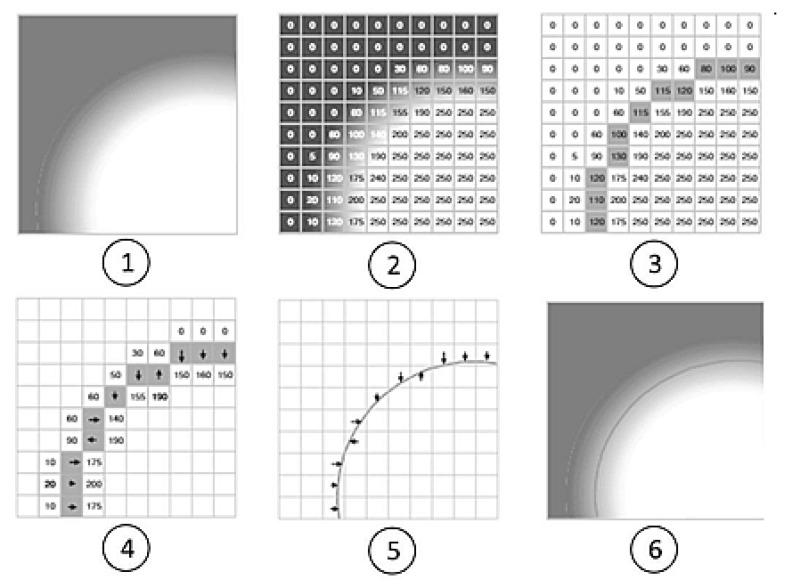
Following steps of the digital outline designation: (**1**) original image; (**2**) digital image; (**3**) pixel contour; (**4**) subpixel contour; (**5**) aligned outline (equation calculus method); (**6**) application of the outline to the original image.

Measurements with the use of Optical Measuring Machines start with mounting the object on the measuring table, setting the appropriate parameters and then performing the measurement which is carried out with the use of a digital camera that takes a digital image of a measured object at programmed locations. After the digital image is taken, an image analysis begins which is aimed at detection of the object edges which can be defined as significant local changes of intensity within the processed image. Most often this process is done using the algorithm that searches for gradient transitions. Then the pixel and subpixel contours are detected which leads to determination of an object outline and its application to the original image [25,26,27,28,29]. The process is presented schematically in Figure 1.

The changes in intensity within an image can be attributed to the geometrical properties of measured objects, but they are also connected with condition under which the digital image was taken such as applied illumination. Techniques used to ensure an appropriate lighting of measured object include: Backlighting, Diffuse Lighting, Direct Incident Lighting or Dark Field Illumination. This paper focuses on the first of mentioned techniques which is well-known for example from its application in microscopy. In this method, the measured part is placed between the sensor and a light source; thus, it is possible to obtain the high contrast between the dark silhouette representing an object shape and a bright background. As the measured part is put in the light beam, the used intensity of illumination can significantly influence measurement results. However, settings connected with the light intensity in Optical CMMs are typically chosen manually by the user. Therefore, a question arises how changes of the lighting intensity within the limits specified by a measuring system manufacturer will affect the measurement errors obtained for certain measuring tasks and the measurement uncertainty.

**Figure 2:**
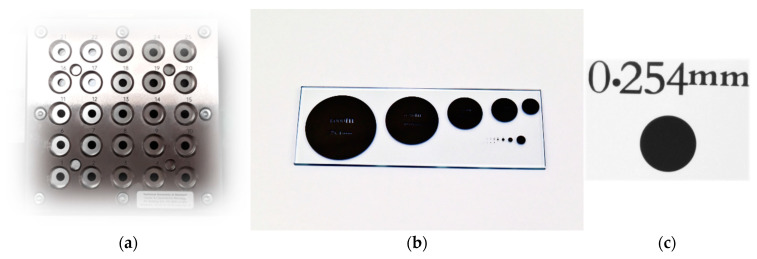
(**a**) Hole plate standard for optical measurements utilized during experiments, (**b**) glass plate standard (circle 254 visible on the left of the second row of circles), (**c**) circle 254 visible in optical coordinate measuring machines (OCMM) optical system.

The above-mentioned problem can be examined by conducting appropriate experiments. One of the methods suitable for this purpose is a methodology based on reference object measurements which are conducted under changing conditions, in this case with changing illumination intensity. The experiment involved measurements of a special hole plate standard for optical systems, shown in Figure 2a, which can be used in a comparison between tactile and mechanical measurements and was described in [30,31]. The second reference object used in research is a reference glass plate with marked circular features of different sizes (depicted in Figure 2b,c). The part coordinate system for the hole plate standard was based on the measurement of three circles whose centers were used for determination of axes of the local coordinate system. Then the zero point of designated datum was moved to the circle number 1. The size of reference object is 80 mm × 80 mm from the center of the circle located in the left bottom of the plate to the circle placed in the top right corner. The circles diameter equal to 5.5 mm, and the distance between circles determined along axes of the local coordinate system equals to 20 mm. The orientation of axes of the local coordinate system for the glass plate was copied from the machine coordinate system; only the origin was set in the center of a chosen circle. The feature used during inspection of this reference object has a diameter of 0.254 mm.

These reference objects were chosen to assess the influence of changing lights properties both on inner and outer dimension measurements. Independent on the type of used reference object, the experiments involved multiple measurements of chosen features with different lighting intensity. After the local coordinate system was determined, chosen circles were measured using 12 points. The initial value of the backlight was determined empirically as the value of L9 which mean that illumination has intensity of 9% of maximum value recommended by measuring system manufacturer. This is the limit number at which the software is able to find the gradient threshold and measure the point. Below this value, the software does not find points and reports an error. The backlight intensities were tested with higher density to value marked as L20, and then, intensities were changed with step 5% up to L100, which means the maximum (100%) available illumination setting on the machine.

In case of hole plate standard, three circles were measured (marked as 1, 13 and 25—see Figure 2), which are placed on the diagonal of the plate. Measurements were repeated 30 times for each applied illumination. After each measurement, the diameter of circle was determined, together with the form deviation and the position of a circle center.

Additionally, position of each measured point was controlled and recorded for each repetition of measuring sequence. They were used in order to determine ellipses of point repeatability. These ellipses can be treated as a quantitative representation of point measurement uncertainty. They were determined in the following way. Firstly, the center of ellipse is calculated using formulas:

(1) $$X=\frac{x_{1}+x_{2}+\cdots +x_{N}}{N}$$

(2) $$Y=\frac{y_{1}+y_{2}+\cdots +y_{N}}{N}$$

where *x_i_*, *y_i_*—coordinates of measured point obtained in subsequent measuring cycles; *N*—number of cycles utilized during experiment (*N* = 30).

Next covariance matrix can be formulated:

(3) $$CM=\left[\begin{array}{cc} \sigma_{X}^{2} & \sigma_{XY}\\ \sigma_{YX} & \sigma_{Y}^{2} \end{array}\right]$$

where $\sigma_{X}^{2}$—variance of first variable *x*; $\sigma_{Y}^{2}—$variance of second variable *y*. $\sigma_{XY}$, $\sigma_{YX}$—the covariance of two variables *x* and *y*.

After these steps, the eigenvalues *λ*_1_. *λ*_2_ of covariance matrix can be calculated, as well as values of eigenvectors *v*_1_, *v*_2_. Then, they are used in order to determine lengths of ellipse’s semi-major (*a*) and semi-minor (*b*) axes with formulas

(4) $$a=2*\sqrt{5.9*\lambda_{1}}$$

(5) $$b=2*\sqrt{5.9*\lambda_{2}}$$

where the eigenvalues *λ*_1_, *λ*_2_ of covariance matrix.

The value of 5.9 is used as a multiplier that is taken from the Chi-square distribution and guarantees a 95% confidence interval.

Finally, the ellipse slope is given as

(6) $$\gamma =\tan^{-1}\frac{v_{1}\left(y\right)}{v_{1}\left(x\right)}$$

where γ—the angle between semi-major axis of ellipse and x-axis of coordinate system; *v*_1_(*x*), *v*_1_(*y*)—components of the largest eigenvector of covariance matrix.

The same experiment procedure was applied for the glass plate but only for one chosen circle.

**Figure 3:**
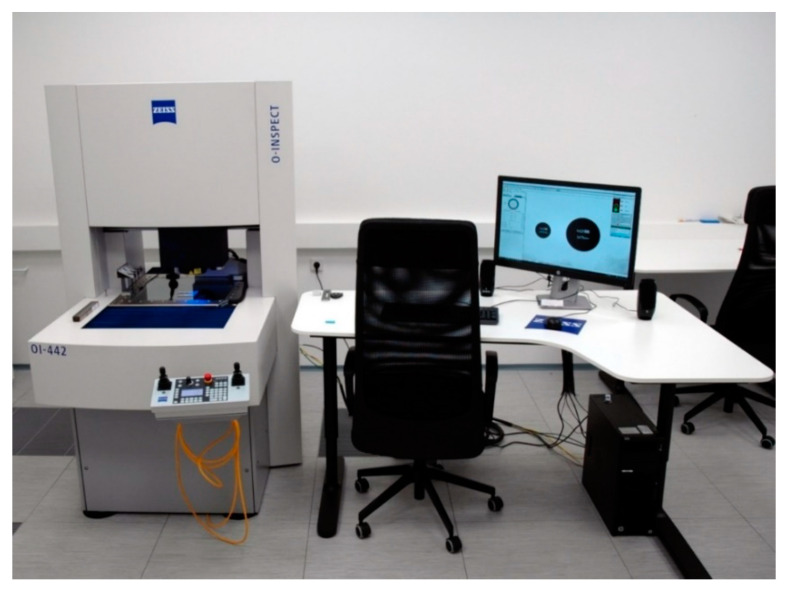
Zeiss O’ Inspect 442 used during experiments, located at the Laboratory of Coordinate Metrology.

All measurements described in the paper were performed at the Laboratory of Coordinate Metrology on an optical multisensor machine Zeiss O’ Inspect 442, presented in Figure 3. The machine is located in an air-conditioned room, and the temperature during the measurements was monitored and changed in the range (20.4; 20.7) °C. Temperature compensation system was turned on in order to minimize thermal influences on measurement results. The accuracy of the machine is described by the equation of maximum permissible errors (7):

(7) MPE = 1.9 + 4 ∗ L/1000 µm

where L is the measured value given in mm.

The largest available magnification, 6.3× and the backlight were used for all measurements included in experiments.

## 3. Results

**Figure 4:**
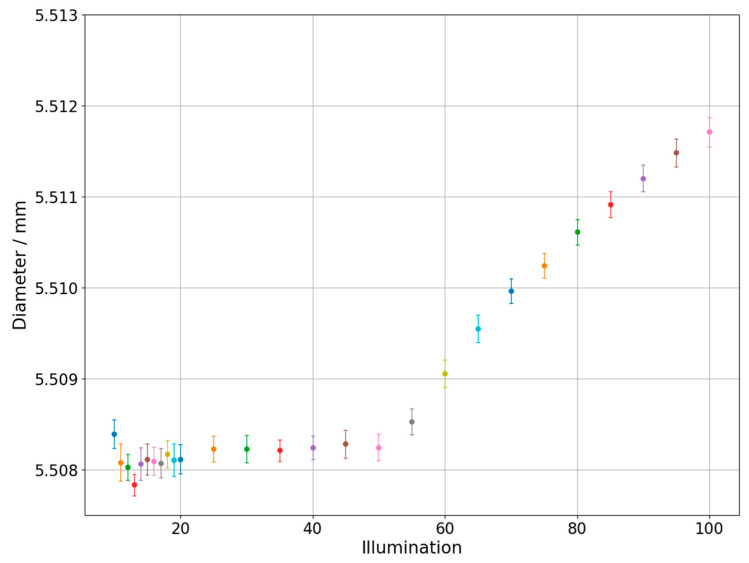
Diameter changes depending on the applied backlight. Hole plate standard measurement.

**Figure 5:**
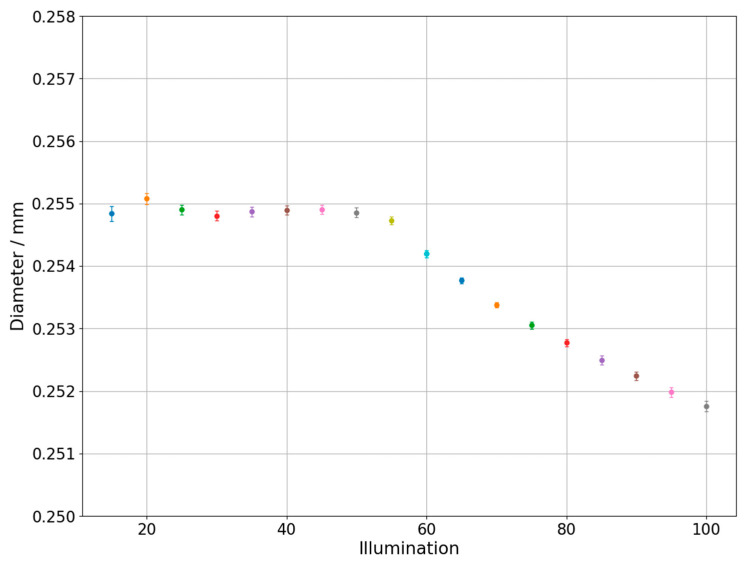
Diameter changes depending on the applied backlight. Glass plate standard measurement.

**Figure 6:**
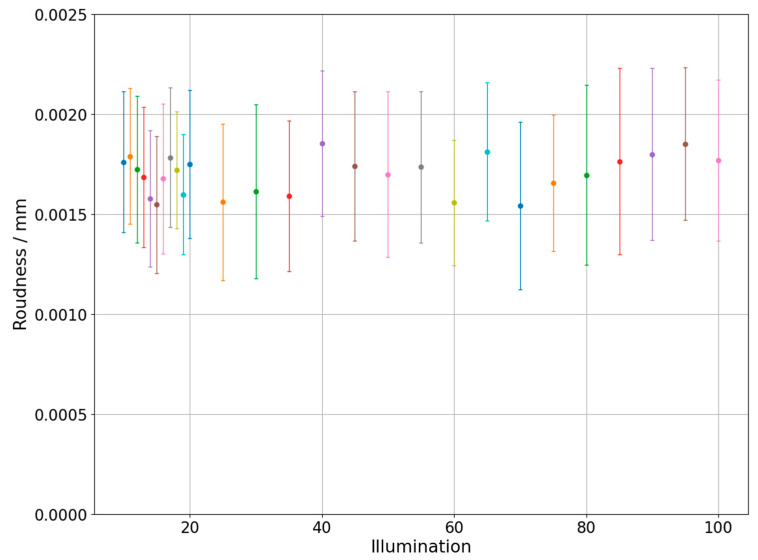
Roundness changes depending on the applied backlight. Hole plate standard measurement.

**Figure 7:**
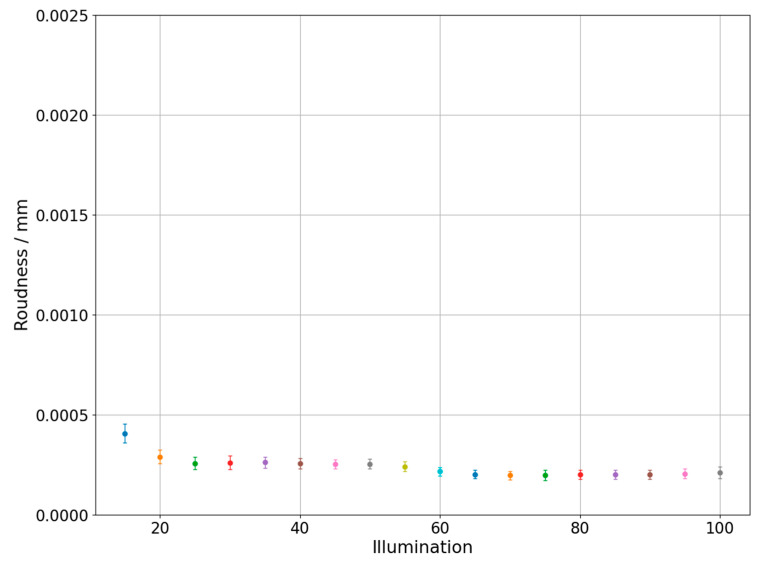
Roundness changes depending on the applied backlight. Glass plate standard measurement.

**Figure 8:**
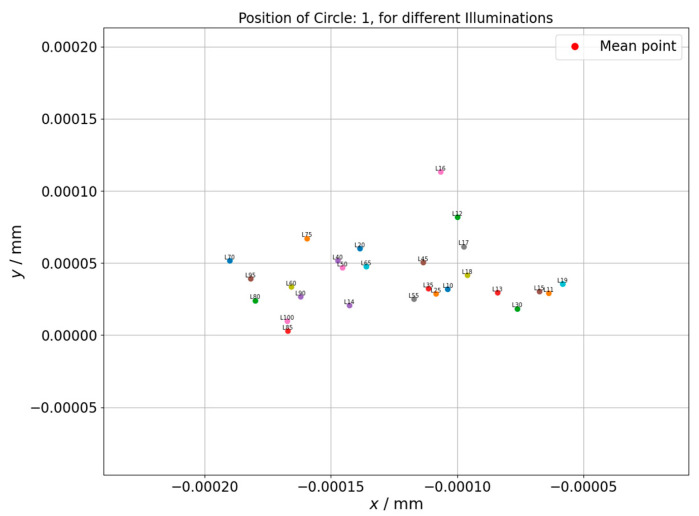
Changes of circle center position depending on the applied backlight. Hole plate standard measurement. Circle 1.

**Figure 9:**
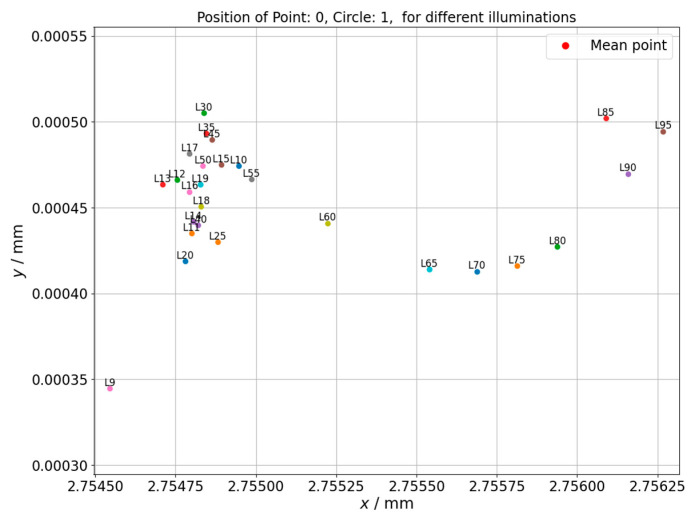
Changes of position of measured point depending on the applied backlight. Hole plate standard measurement. Circle 1.

Changes of measured diameter depending on the utilized backlight are presented in Figure 4 which was prepared for the one of the circles on the hole plate and in the Figure 5 which represents results obtained during measurements of circle with diameter 0.254 mm marked on the glass plate. The different colors of dots and error bars in Figure 4, Figure 5, Figure 6, Figure 7, Figure 8 and Figure 9 have no additional meaning, they are used in order to improve legibility of presented results. The error bars in Figure 4, Figure 5, Figure 6 and Figure 7 show the standard uncertainty values of the diameter/roundness determination.

In case of inner dimension, the values on the left side of the graph corresponding to the lowest backlight values are rather unstable. This may be due to insufficient lighting of the tested element, which results in the erroneous detection of the edge and, consequently, erroneous acquisition of measured point. For backlight values from approximately 20 to 50, the characteristic is close to straight line without major deviations. However, from the value of 50, a significant change in the value of the diameter is noticeable, which progresses with the increase of the backlight. Such a characteristic is interesting because from the analysis of literature and from the point of view of the physical properties of light, a linear characteristic should be expected in the whole range of tested lighting parameters. Similar characteristic was obtained in case of glass plate standard, but the increase in lighting caused a decrease of the measured diameter instead of increase which was observed for hole plate. Additionally, measurements with lower values of applied backlight show higher stability than in previous case.

The next figures (Figure 6 and Figure 7) show changes of roundness error depending on the applied backlight value. Figure 6 shows results obtained during hole plate measurements, while Figure 7 presents outcome of glass plate measurements.

Similarly, to the analysis of diameter changes for hole plate measurements, the initial values show lower stability, especially the value obtained for the L9 backlight. As in the previous case, the problem is probably a small light intensity and, consequently, a big variability given by calculation algorithms. The next part of the graph does not show the dependence of the error value with the increasing backlight. Moreover, measurements of glass plate showed that changing backlight does not significantly affect roundness measurements. On the other hand, the difference of approximately 0.0015 mm in measured roundness can be observed between presented figures. Considering that both reference objects have small form deviations such observation is rather surprising. It may be connected with the size of measured features. In case of glass plate, the whole circle can be analyzed on the basis of one digital image. For bigger features, it is necessary to measure circle partially, so the measurement accuracy is prone to additional error sources.

The results of the measurement of the center point position for the circle 1 of the hole plate are shown in the Figure 8. The graph presents how the center position differs depending on applied backlight.

The center point position for the circle 25 of the hole plate was also measured. It was observed that for both measured circles, the experiment provided similar results. In both cases, the dispersion of circle positions in x axis is within 0.1 µm and in y axis within 0.1 µm for the circle that is closer to the hole plate coordinate system and within 0.2 µm for the one that is farther. This level of changes should rather be attributed to random influences, especially that no clear upward or downward trend is observed. More unambiguous results were obtained from controlling the position of individual measured points, where the point position is not influenced by averaging algorithms like best fit that was used for circles determination. The next figure (Figure 9) shows the changes of position of one of controlled points (north pole of the circle) depending on applied illumination intensity. 

The same change was also investigated for a point in the north pole of another circle (circle 25). Similar results were obtained for both considered circles. The higher illumination intensity results in shift of measured point position in positive direction of x axis which can be related with the blurring of the edges due to the greater intensity of the applied lighting.

**Figure 10:**
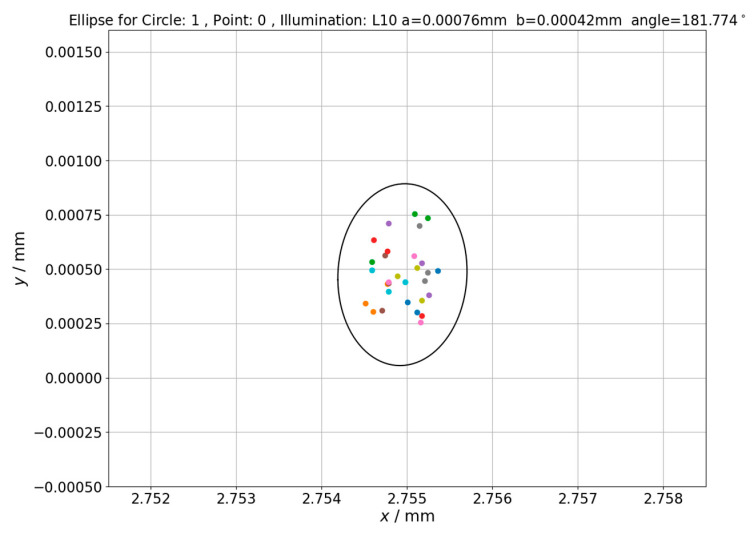
The ellipse of point repeatability obtained for first point in measurement routine. Hole plate standard measurements—Circle 1. Applied illumination L10.

**Figure 11:**
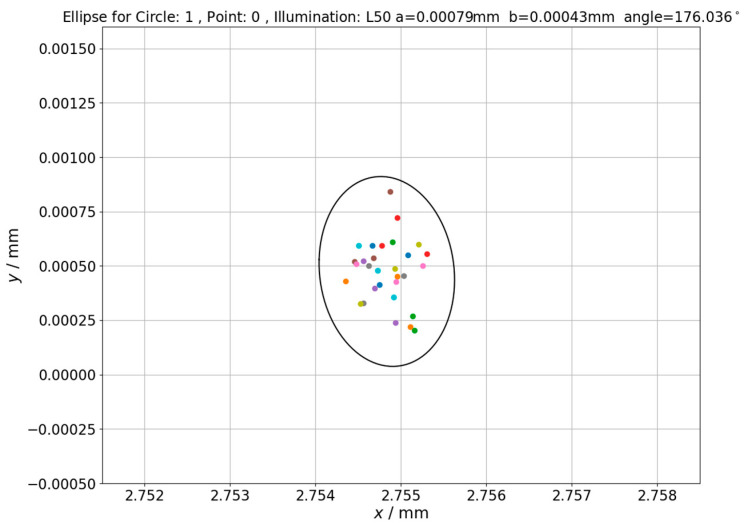
The ellipse of point repeatability obtained for first point in measurement routine. Hole plate standard measurements—Circle 1. Applied illumination L50.

**Figure 12:**
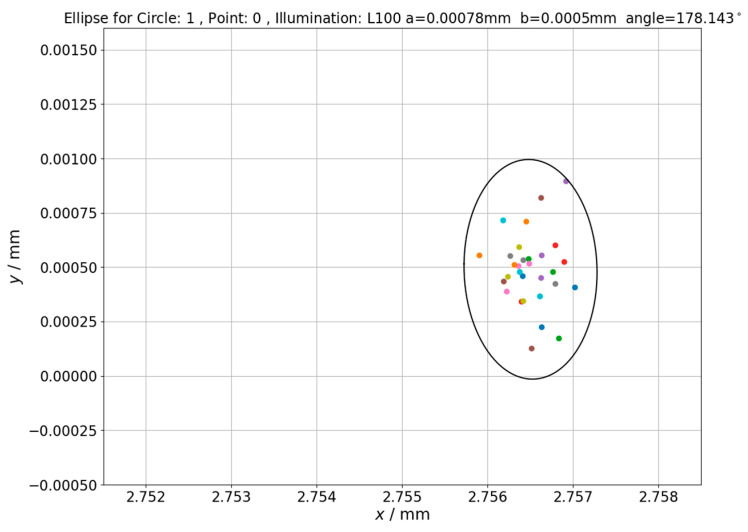
The ellipse of point repeatability obtained for first point in measurement routine. Hole plate standard measurements—Circle 1. Applied illumination L100.

The influence of changing backlight intensity on measurement uncertainty was checked in last part of experiment which involved determination of ellipses of point repeatability (see Section 2 for further details). The next Figures (Figure 10, Figure 11 and Figure 12) shows ellipses obtained for first measured point in inspection routine for different circles measured on hole plate standard.

Analyzing the obtained results, it should be noted that increase of lighting intensity affected the area of determined ellipses but not significantly (quantitative data related to this issue is given in next section). On the other hand, for the highest presented illumination value, the shift of ellipse center is clearly visible which should be attributed to illumination effect on position of measuring point. Values of ellipses’ centers translation along x axis corresponds to values of measuring point position changes presented in Figure 10 and Figure 11. The lengths of ellipse’s semi-major (a) and semi-minor (b) axes for other ellipses of point repeatability determined within presented research are summarized in Table 1.

## 4. Discussion

The presented research aimed to determine:

- Values of task-specific error changes related to a measurement of distances, positions and circularity deviation under changing lighting conditions.
- Changes in uncertainty areas of a measuring point repeatability for point measurements under changing lighting conditions.

General conclusion coming from analysis of performed experiments results is that they show quite significant effect of the backlight on the results of circles’ diameters and points’ positions measurements (after exceeding a certain level of illumination, which is L50 for considered OCMM); on the contrary, effects of illumination on results of circularity deviation measurements are negligible and small on uncertainty of point measurement.

It is also worth noting that a location of symmetrical features (like circular holes) is rather insensitive to effects that perturb edge detection, while size determinations that do not benefit from the symmetry of the hole are much more strongly affected. The effects which are unimportant when measuring location of centers of symmetric features can become a serious problem for less-symmetrical ones because the effect of illumination change on determination of the feature’s edges may not be the same for sections of the feature with different, unsymmetrical shapes.

Other general observations are that the detected single point positions may change by several micrometers over a range of reasonable intensity choices (this change was not bigger than 3 µm for presented research) and that the shift is non-linear. Presented results also show that this change can depend on the position relative to the machine coordinate system.

As can be seen on Figure 4 and Figure 5, the magnitude of illumination influence on both external and internal diameter measurements is similar. The difference is that for measurements of internal diameter with the increase of illumination the diameter rises while for measurements of external diameter it declines. This finding is consistent with results obtained by other researchers in case of measurements performed on optical systems different than OCMM.

Below, the correction function is proposed. It presents the value of systematic error of internal circle diameter measurement that may be attributed to application of illumination level above the L50 level during the measurement. Firstly, it is explained how this function is built and how it was obtained on the example of research performed within this paper. As such, functions are specific to the measuring machine that was used (results presented in this paper are valid only for OCMM used during described experiments); in the next step, the general procedure of determination of this kind of functions on any OCMM is presented.

The influence of illumination changes above L50 level on results of internal circle’s diameter measurements was approximated using polynomial function. Approximation of experimental data was performed using linear, polynomial (with maximum function degree equal to 3), exponential and logarithmic functions; the best approximation result was chosen by calculation of the coefficient of determination (R^2^) and selection of the function for which value of this coefficient was highest (R^2^ was bigger than 0.98 for all cases). In the presented case, the polynomial function was selected and may be given as (8):

(8) $$\begin{array}{c} \Delta d\left(I_{\mathrm{ratio}}\right)=-163.170*\;10^{-5}{*I}^{3}+128.205*10^{-5}{*\;I}^{2}+501.398*10^{-5}*I\\ -8.881*10^{-5}\;\mathrm{mm} \end{array}$$

where Δd is a value of diameter of circle change that may be taken as an approximation of systematic error attributed to illumination level change, I_ratio_ is calculated using following Equation (9):

(9) $$I_{\mathrm{ratio}}=\frac{I-I_{\mathrm{smax}}}{I_{\max}-I_{\mathrm{smax}}}$$

where I is the illumination level applied during measurements (in the presented case, it may change in the range of (L50; L100>); I_smax_ is the last illumination level for which the results of diameter measurements are stable (in the presented case it is L50); I_max_ is the maximum illumination level that may be used during measurements (in the presented case it is L100).

For the function presented in (8), the mean approximation error equaled to 0 mm, and the maximum approximation error was not bigger than 0.00013 mm.

In order to be able to easily calculate the value of the systematic error of circle diameter measurement that may be attributed to a certain illumination level directly from function (8), the mean diameter value determined from measurements performed for illumination in the range from L15 to L50 (where the measurement results are stable) was deducted from diameter values obtained for illuminations set for values bigger than L50.

Similar influence functions may be determined for an external circle’s diameter and, based on results of point position measurements, for systematic errors of point coordinates that determine the measuring point position.

The general procedure of determination of this kind of functions on any OCMM is as follows:

1. Perform experiments as described in Section 2 of this paper.
2. Determine the range in which measurement results are stable. Determine the I_smax_ as a last illumination level for which the results are stable. Calculate mean value of measurement results in stability range (if changes of considered characteristic values are visible for all illumination levels, without stability range, omit this step and the next one).
3. Deduct mean value determined in step 2 from considered characteristic values obtained for illuminations set for values bigger than I_smax_.
4. For all illumination levels above I_smax_ (if step 3 was omitted take minimum illumination applied during measurements as I_smax_) calculate I_ratio_ using (9).
5. Determine influence functions that give the relation between I_ratio_ values and values determined in step 3 (or of rough measurement results if step 3 was omitted) using different approximation methods (for example linear, polynomial, exponential and logarithmic approximation, or any other relevant approximation method).
6. Calculate the coefficient of determination (R^2^) and select the function (out of functions determined in step 5) for which the value of this coefficient is the highest.

The next issue that was investigated was changes in uncertainty areas of the measuring point reproduction for point measurements performed under changing lighting conditions. Results of these investigations were presented in Figure 10, Figure 11 and Figure 12. Visual analysis of these figures shows that increase of lighting intensity does not significantly affect the area of determined ellipses. In order to quantify changes in uncertainty areas, ellipses fields (*S*) determined using Equation (10) are presented in Table 1.

(10) S=πab

Results presented in Table 1 show that changes in uncertainty areas of measuring point reproduction caused by different illumination level applied during measurement reach 30% (estimated separately for each point). No clear rising or declining tendency is however observed with increase of illumination level.

Since many metrological programs used during optical measurements do not have the possibility of automatic backlight selection, the conducted experiment can be useful to increase the attention of users to the selection of appropriate backlight during measurements. Analysis of the results showed that there is a limit value (L50) for a particular software for which the results behave stably, but after exceeding it, the measurement error increases noticeably. It was also shown that for some measuring tasks (measurement of internal circles) applying too low illumination may also cause instabilities in obtained results, which may be attributed to worse performance of algorithms responsible for detection of the edges of measured objects.

Illumination influences on results of measurements performed on OCMM equipped with video probe that were determined in this paper are valid only for the CMM that was used during presented experiments; however, the described methodology may be applied to other OCMMs and similar influence equations may be established for them.

Influence functions determined within this research will be used in the virtual model of OCMM that is now in the final stage of development for determination of a single measurement systematic error. Information regarding possible level of illumination influence on the uncertainty of measuring point reproduction will be used for scaling the uncertainty ellipses obtained from Monte Carlo simulations for points being the subject of simulations. The fully functional virtual model of OCMM will be described in future publications.

## Figures and Tables

**Table 1:** Changes in areas of measuring point reproduction ellipses (*S*) caused by application of different illumination level during point measurements.

Ellipse Determined for	Illumination Level	*X*/mm	*Y*/mm	*a*/mm	*b*/mm	*S*/mm^2^
Point 0, circle 254	L10	0.12705	−0.00046	0.00127	0.00016	6.384 ∗ 10^−7^
	L50	0.12712	−0.00046	0.00125	0.00016	6.283 ∗ 10^−7^
	L100	0.12667	−0.00045	0.00126	0.00016	6.333 ∗ 10^−7^
Point 0, circle 1	L10	2.75495	0.00047	0.00076	0.00042	10.028 ∗ 10^−7^
	L50	2.75483	0.00047	0.00079	0.00043	10.672 ∗ 10^−7^
	L100	2.75650	0.00049	0.00078	0.00050	12.252 ∗ 10^−7^
Point 0, circle 13	L10	42.75819	39.99901	0.00071	0.00056	12.491 ∗ 10^−7^
	L50	42.75812	39.99894	0.00080	0.00052	13.069 ∗ 10^−7^
	L100	42.75972	39.99892	0.00089	0.00042	11.743 ∗ 10^−7^
Point 0, circle 25	L10	82.75978	79.99133	0.00093	0.00042	12.271 ∗ 10^−7^
	L50	82.75981	79.99130	0.00063	0.00051	10.094 ∗ 10^−7^
	L100	82.76230	79.99128	0.00067	0.00051	10.735 ∗ 10^−7^

## Data Availability

The data presented in this study are available on request from the corresponding author.
